# MiR‐21 is up‐regulated in urinary exosomes of chronic kidney disease patients and after glomerular injury

**DOI:** 10.1111/jcmm.14317

**Published:** 2019-05-07

**Authors:** Tim Lange, Nadine Artelt, Frances Kindt, Sylvia Stracke, Rainer Rettig, Uwe Lendeckel, Christos E. Chadjichristos, Panagiotis Kavvadas, Christos Chatziantoniou, Karlhans Endlich, Nicole Endlich

**Affiliations:** ^1^ Department of Anatomy and Cell Biology University Medicine Greifswald Greifswald Germany; ^2^ Department of Internal Medicine A University Medicine Greifswald Greifswald Germany; ^3^ Institute of Physiology, University Medicine Greifswald Karlsburg Germany; ^4^ Department of Medical Biochemistry and Molecular Biology University Medicine Greifswald Greifswald Germany; ^5^ Unité Mixte de Recherche (UMR)‐S1155 Tenon Hospital, National Institute for Health and Medical Research (INSERM), Sorbonne Universités Paris France

**Keywords:** biomarker, CKD, miR‐21, miRNA, urinary exosomes

## 1 INTRODUCTION

With 500 million people affected worldwide, chronic kidney disease (CKD) constitutes a major public health problem.^1^ Mostly arising from arterial hypertension and diabetes mellitus, it is a terminal disease without any causal therapy, leading to dialysis and/or kidney transplantation. Glomerulopathies are the main cause of end stage renal disease (ESRD), which are mainly caused by podocyte effacement or loss.^2^

A relevance of microRNAs (miRs) on podocyte function and the pathogenesis of glomerulopathies^3^ could have been clarified. Both, their molecular influence on translation and their potential role as biomarkers for kidney diseases, including CKD, became of major research interest. Especially miRs derived from urinary exosomes appear to have a promising potential as biomarkers, since they can be accessed non‐invasively and they are protected against degradation,^4^ making sample preparation more unsusceptible against environmental influences. Unfortunately, only little is known about exosomal miR expression in urine of CKD patients.

Therefore, the present study aims at the investigation of the expression levels of the well‐investigated miRs miR‐21, miR‐30a‐5p and miR‐92a in urinary exosomes of CKD patients, representing a new promising, non‐invasive sample type and their functional roles in different injury models.

## 2 MATERIAL AND METHODS

### 2.1 Patient urine samples

Patient urine samples were obtained within the scope of the Greifswald Approach to Individualized Medicine (GANI_MED).^5^ A written informed consent was signed by all participants. The study followed the ethical rules of the declaration of Helsinki. For a precise description see Data S1.

### 2.2 Urinary exosomal miRNA isolation, reverse transcription and RT‐qPCR

Urine processing, exosome preparation, miR isolation and reverse transcription quantitative polymerase chain reaction (RT‐qPCR) were performed as described previously.^6^ Taqman™ miRNA Assays: See Data S1. For a precise description see Data S1.

### 2.3 Glomeruli dedifferentiation assay, RNA isolation and RT‐qPCR

The isolation, cultivation and RNA isolation of murine glomeruli was performed as described by Kindt et al.^7^ Taqman™ miRNA Assays: See Data S1. For a precise description see Data S1.

### 2.4 Nephrotoxic serum treatment, RNA isolation and RT‐qPCR

Mice handling and nephrotoxic serum (NTS) treatment were performed as previously described.^8^ All mouse experiments were performed in accordance with the national animal protection guidelines that conform to the National Institutes of Health Guide for the Care and Use of Laboratory Animals and were approved by the local governmental authorities. For a precise description see Data S1.

### 2.5 Statistical analysis

See Data S1.

## 3 RESULTS

### 3.1 Patient characteristics

The patient set consisted of 41 patients suffering from CKD and five healthy controls. CKD patients had a mean age of 47 (±10) years and healthy controls had a mean age of 40 (±15) years. There were 17 females in the CKD group and three in the healthy control group. In the CKD group 30 individuals exhibited diabetes mellitus, which was defined as HbA1C values of ≥6.5%. The mean urinary albumin to creatinine ratio (UACR) was 1598.7 (±2297.5) mg/g in CKD patients. The mean estimated glomerular filtration rate (eGFR) was 22.5 (±18.7) mL/min/1.73 m^2^in the CKD group. For the healthy control group we did not collect data regarding UACR and eGFR (Table S1).

### 3.2 Levels of miR‐21 are enhanced in urinary exosomes of CKD patients

**Figure 1:**
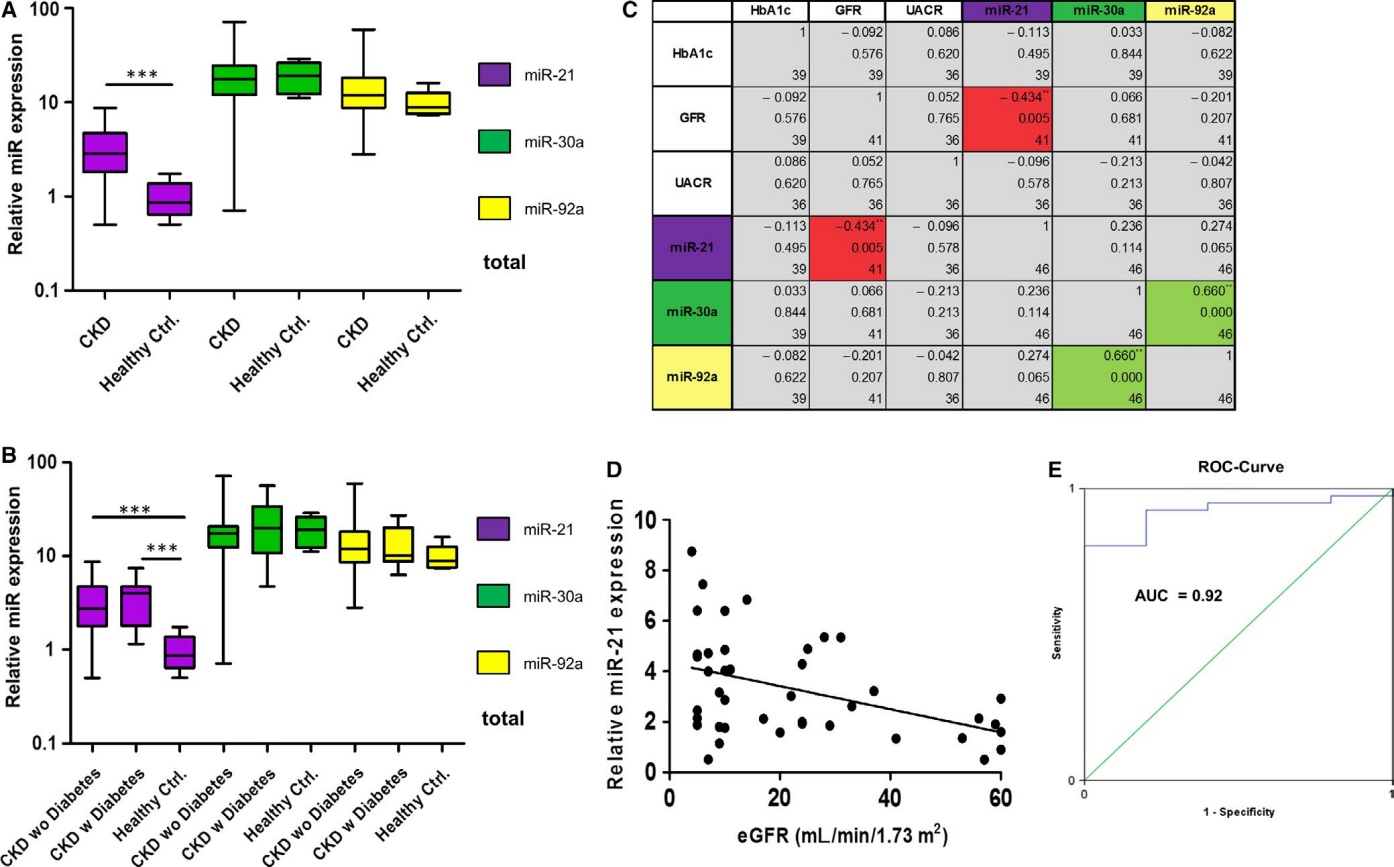
Levels of urinary exosomal miR‐21, miR‐30a‐5p and miR‐92a in chronic kidney disease (CKD) patients and healthy controls. MiR levels were measured by RT‐qPCR normalized against inter‐run calibrator and miR‐16. MiR‐21 is significantly up‐regulated in CKD patients. *y*‐axis = Log_10_, error bars = SD (A). There is no difference between CKD patients with or without diabetes *y*‐axis = Log10, error bars = SD (B). MiR‐21 is negatively correlated with eGFR (red boxes). MiR‐30a‐5p is positively correlated with miR‐92a (green boxes) (C,D). Boxes top down: *R* (correlation coefficient), *P*‐value, n. *P ≤ 0.05, **P ≤ 0.01, ***P ≤ 0.001 (C), *y*‐axis = Log_2_ (D). ROC‐curve analysis of miR‐21. Green line = diagonal; blue line = ROC‐curve; AUC, area under the curve (E)

We found a significantly (*P* < 0.001) higher average level of miR‐21 in CKD patients (3.3 ± 1.9) compared to healthy controls (0.9 ± 0.5) (Figure 1A). Only three CKD patients had a lower miR‐21 level than the average level of healthy controls (Figure S1). We also found significantly (*P* = 0.001, *P* < 0.001, respectively) higher average levels of miR‐21 in CKD patients with (3.5 ± 1.9) and without diabetic nephropathy (DN) (3.2 ± 2.0) compared to healthy controls (0.9 ± 0.5) (Figure 1B). Receiver operating characteristic (ROC) analysis (ROC) revealed an area under the curve of 0.92 (Figure 1 E). We did not observe significant differences between CKD patients and healthy controls with regard to miR‐30a‐5p and miR‐92a, respectively (Figure 1A,B).

### 3.3 MiR‐21 is negatively correlated with eGFR

We found a statistically significant positive correlation between miR‐30a‐5p and miR‐92a of 0.660 (*P* < 0.001). The only statistically significant correlation (*P* = 0.005) between clinical parameters HbA1c, eGFR and UACR and miR expression levels we detected, was a negative correlation between miR‐21 and eGFR of −0.434 (Figure 1C,D).

### 3.4 MiR‐21 expression is up‐regulated in (de‐)differentiated glomeruli

**Figure 2:**
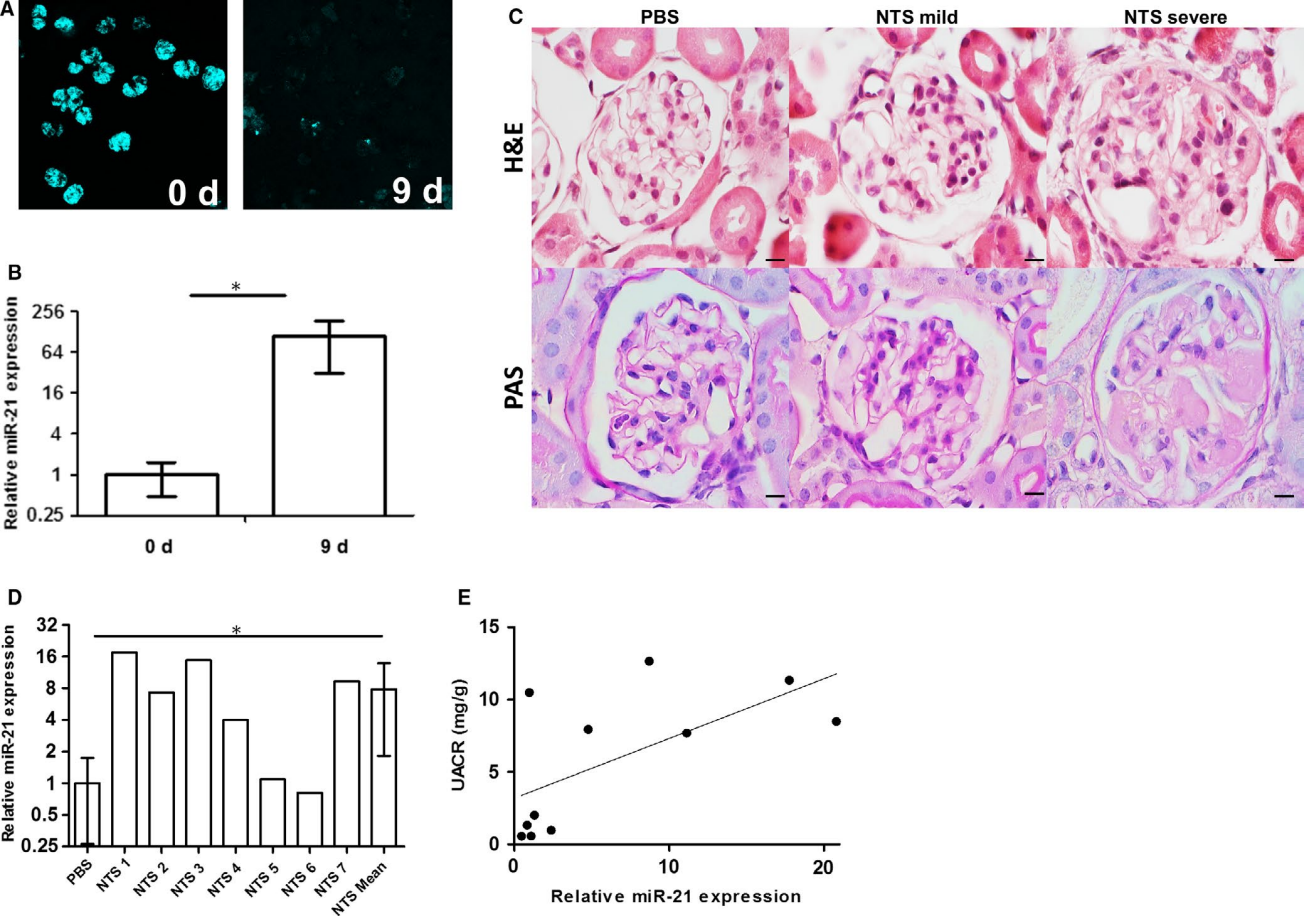
MiR‐21 levels in dedifferentiated podocytes and in nephrotoxic serum (NTS)‐treated mouse kidneys. Dedifferentiated glomeruli show almost no cyan fluorescent protein fluorescence (A). MiR‐21 is 108.8 times up‐regulated in dedifferentiated glomeruli. *y*‐axis = Log_2_; error bars = SD; n = 3. (B) The glomeruli of NTS treated mice show two main phenotypes in H&E and PAS staining. Scale bars = 10 µm. (C). MiR‐21 is 8.9 times up‐regulated in NTS‐treated formalin‐fixed paraffin‐embedded (FFPE) mice kidneys. *y*‐axis = Log_2_; error bars = SD; PBS n = 4; NTS n = 7. (D). Pearson correlation of relative miR‐21 expression and UACR. *x*‐axis = Log_10_ (E)

Freshly isolated glomeruli showed a strong cyan fluorescent protein (CFP) signal in all glomeruli driven by a nephrin promoter fragment. In glomeruli cultured for 9 days the CFP signal almost totally disappeared (Figure 2A). Glomeruli cultured for 9 days showed a statistically significant enhanced relative miR‐21 expression of 108.8 (±78.0) compared to 1.0 (±0.5) in freshly isolated glomeruli (*P* = 0.037) (Figure 2B). Interestingly, dedifferentiated glomeruli also showed an up‐regulation of genes involved in MAP‐ERK signalling (Data S1).

### 3.5 MiR‐21 is overexpressed in NTS‐treated mice

Nephrotoxic serum‐treated mice showed development of glomerulonephritis as shown by Hematoxylin and eosin (H&E) staining and Periodic acid–Schiff (PAS) staining (Figure 2C). We could observe glomeruli with two main phenotypes. The more abundant mild phenotype displayed glomerular basement membrane accumulation and mesangial matrix expansion, whereas the severe phenotype displayed crescent formation and podocyte loss. The PBS‐injected control mice showed a normal histological morphology. Interestingly, we could find a significant (*P* = 0.035) average 8.9‐fold up‐regulation of miR‐21 in mice injected with NTS in relation to PBS‐injected control mice (Figure 2D). The miR‐21 expression was correlated with UACR (*R* = 0.632; *P* = 0.037) as shown in Figure 2E.

## 4 DISCUSSION

Besides conventional biomarkers, urinary miRs display a promising non‐invasive tool as potential biomarkers for CKD. In contrast with total urine, urine sediment or cell‐free urine samples, miRs derived from urinary exosomes are protected against degradation by nucleases and are protected against other environmental factors potentially compromising RNA integrity.^4^ This makes them a new sample type with a promising potential in simplifying the handling and storage of patient urine samples.

Most of the current studies performed total miR profiling on relatively small patient sets or concentrated on a single miR in a specific disease. In the present study, we used a randomly picked, widespread set of patients suffering from CKD.

We could not find significant differences in expression levels between CKD patients and healthy controls with regard to miR‐30a‐5p. Interestingly, miR‐30a‐5p was recently proposed as a potential biomarker for FSGS in human urine samples.^9^ Since our cohort represents a randomly picked patient set, it is likely that it is composed of FSGS patients to a statistically insufficient number. This might also be the case for miR‐92a, which is known to be up‐regulated in crescentic rapid progressive glomerulonephritis.^10^

In the present study, we found miR‐21 being significantly up‐regulated in urinary exosomes of patients suffering from CKD. However, miR‐21 expression cannot discriminate between CKD patients with or without diabetes. Even though miR‐21 was not correlated with UACR, which can be explained by high within‐person variability,^11^ it was found to be negatively correlated with eGFR indicating a possible opposing effect on kidney function.

Our results are in agreement with those of other groups describing that miR‐21 is up‐regulated in biopsies and different body fluids of patients suffering from diverse kidney diseases 12, 13 including CKD.

Since podocyte dedifferentiation is a main event in the pathogenesis of glomerulopathies,^14^ we studied the expression of miR‐21 in our glomeruli dedifferentiation assay.^7^ Here, we found a strong up‐regulation of miR‐21 during the dedifferentiation of mouse podocytes during 9 days and an up‐regulation of genes involved in MAP‐ERK signalling as described before.^15^

Moreover, we applied NTS to mice that induces glomerulonephritis as an CKD injury model.^16^ We found a strong up‐regulation of miR‐21, underlining the important role of miR‐21 in glomerular pathogenesis.

Summarizing, we found an up‐regulation of miR‐21 urinary exosomes of CKD patients and in different podocyte injury models, indicating that urinary exosomal miR‐21 could be used as a potential new non‐invasive, prognostic biomarker for CKD.

## CONFLICT OF INTEREST

There are no conflicts of interest.

## AUTHORS' CONTRIBUTIONS

TL and NE did the study design. SS and TL contributed to urine processing. TL performed the urinary exosomal miRNA experiments including statistical analysis. FK and TL performed the experiments regarding the (de‐)differentiation assay. TL, NE and KE analysed the experimental data. NA, TL, PK, CEC, CC did the NTS treatment experiments. TL did the figure design and literature search. TL, NE and KE wrote the main manuscript text. RR and UL contributed to manuscript revisal and to the GANI_MED study. All authors had approved the final manuscript.
