# Supplementary material for: MiR‐21 is up‐regulated in urinary exosomes of chronic kidney disease patients and after glomerular injury
Source: J Cell Mol Med. 2019 May 7;23(7):4839–43. doi: 10.1111/jcmm.14317 (PMC6584549; doi:10.1111/jcmm.14317)
Supplement: Supplementary file 1 [file JCMM-23-4839-s001.docx]

S1

**TAQMAN-ASSAYS**

**Urine exosomes:**

hsa-miR-16-5p: ID #000391; hsa-miR-21-5p: ID #000397; hsa-miR-30a-5p: ID #000417; hsa-miR-92a-3p: ID #000431

**Glom-Assay, NTS:**

Hsa-miR-21-5p: ID #000397; U6 snRNA: ID #001973

**Cycler scheme:**

10 min at 95°C followed by 45 cycles of 15 sec at 95°C and 60 sec at 60°C.

**PRECISE METHODS**

**Patient urine samples**

We got no urine albumin-to-creatinin ratio (UACR) values for 5 of the 41 CKD patients, but considered this in the data analysis. Urine from healthy individuals was provided by 5 volunteers from the Department of Anatomy and Cell Biology of the University Medicine Greifswald.

**Urinary exosomal miRNA isolation, reverse transcription and RT-qPCR**

RT primers were pooled to equal amounts. qPCR was performed on the Bio-Rad iCycler Thermal Cycler with the iQ5 Multicolor Real-Time PCR Detection System (Bio-Rad, Hercules, CA, USA). Data analysis was performed by the Bio-Rad iQ5 2.1 software with manually set thresholds and baselines. All Ct-values were inter-run-calibrator corrected and were normalized against miR-16 [6].

**Glomeruli dedifferentiation assay, RNA isolation and RT-qPCR**

RNA from freshly isolated and 9-days-cultured glomeruli was used for analysis. RT-qPCR was performed as described above with 10 ng of total RNA. All Ct-values were normalized against U6 snRNA.

**Nephrotoxic serum treatment, RNA isolation and RT-qPCR**

Four mice were injected with PBS (control) and 7 mice were injected with NTS. After 12 days, urine samples for UACR measurement were taken and mice were sacrificed. RNA was isolated from formalin-fixed paraffin-embedded (FFPE) sections as previously described [9] with some changes. We used two 10 µm thick slices to optimize de-paraffination. Proteinase K digestion was performed for 20 min. We 3-5 ceramic beads to the tissue samples supplemented with Trizol for better tissue disruption. RT-qPCR was performed as described above with 1 ng miR. Raw Ct-values were normalized against U6 snRNA.

**Statistical analysis**

Statistical analysis was performed using the IBM SPSS Statistics 22.0 software (SPSS Inc., Chicago, IL, USA). Data was tested for normality by Kolmogorov-Smirnov test and with Student`s t-test for differences between two groups. The two-tailed tests with p-values ≤ 0.05 were considered statistically significant.

**Table 1. Patient characteristics of study participants.** Number (n) of participants, Sex, Mean age in years and standard deviation (±), Number and percentage of diabetics, urinary creatinine to albumin (UACR) and estimated glomerular filtration rate (eGFR) are shown.


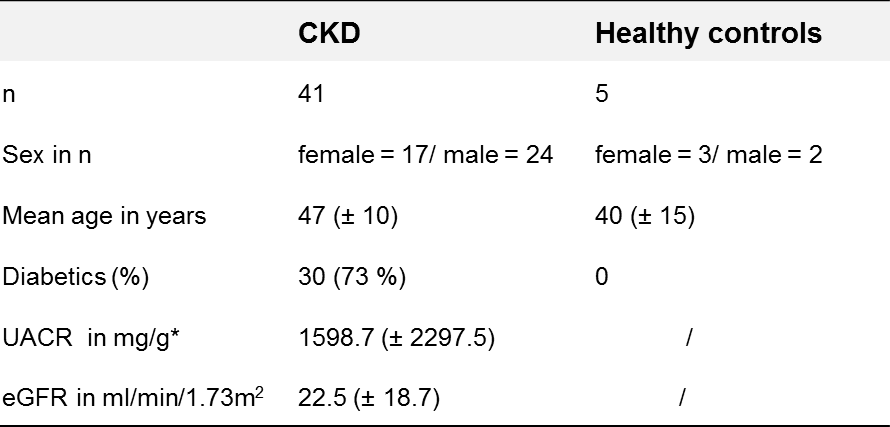


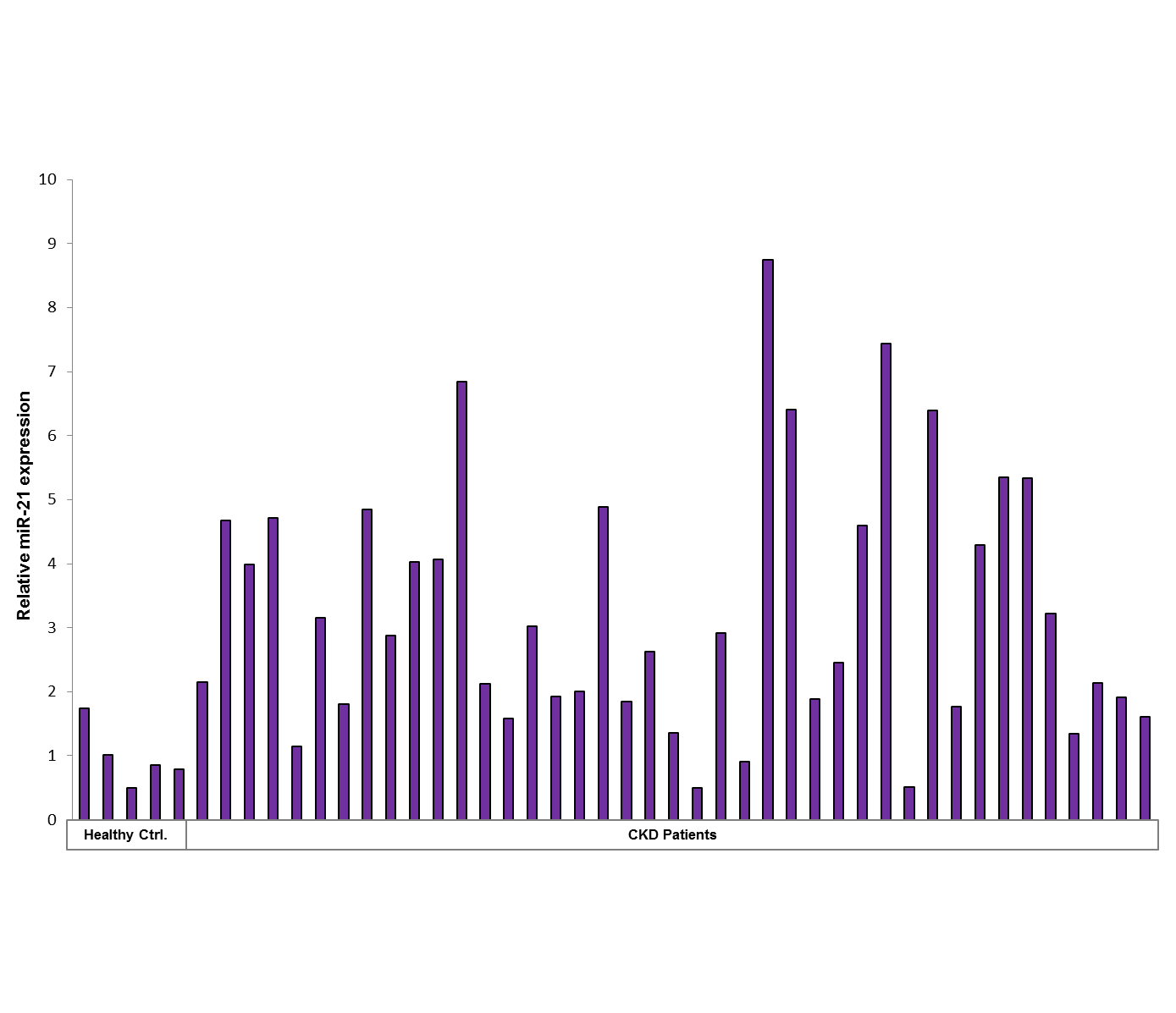
 **Figure S1: Relative miR-21 expression.** The expression of miR-21 is shown in single healthy control probants (Healthy Ctrl.) and single CKD patients (CKD Patients). Expression is normalized to miR-16 and the inter-run calibrator.

**Regulated MAP-ERK Genes**

**NGS:**

Mapk1 117%

Map2k1 147%

Map2k2 119%

Map3k2 151%

**LC-MS:**

Mapk1 110%

Map2k1 196%
